# Remote Blood Pressure Monitoring With a Wearable Photoplethysmographic Device (Senbiosys): Protocol for a Single-Center Prospective Clinical Trial

**DOI:** 10.2196/30051

**Published:** 2021-10-07

**Authors:** Sara Schukraft, Assim Boukhayma, Stéphane Cook, Antonino Caizzone

**Affiliations:** 1 Department of Cardiology University and Hospital Fribourg Fribourg Switzerland; 2 Microcity Pôle d'innovation Neuchâtel Neuchâtel Switzerland

**Keywords:** continuous blood pressure monitoring, photoplethysmography, arterial line, Senbiosys, wearable devices, blood pressure, remote monitoring, continuous monitoring, mHealth, mobile health

## Abstract

**Background:**

Wearable devices can provide user-friendly, accurate, and continuous blood pressure (BP) monitoring to assess patients’ vital signs and achieve remote patient management. Remote BP monitoring can substantially improve BP control. The newest cuffless BP monitoring devices have emerged in patient care using photoplethysmography.

**Objective:**

The Senbiosys trial aims to compare BP measurements of a new device capturing a photoplethysmography signal on the finger versus invasive measurements performed in patients with an arterial catheter in the intensive care unit (ICU) or referred for a coronarography at the Hospital of Fribourg.

**Methods:**

The Senbiosys study is a single-center, single-arm, prospective trial. The study population consists of adult patients undergoing coronarography or patients in the ICU with an arterial catheter in place. This study will enroll 35 adult patients, including 25 patients addressed for a coronarography and 10 patients in the ICU. The primary outcome is the assessment of mean bias (95% CI) for systolic BP, diastolic BP, and mean BP between noninvasive and invasive BP measurements. Secondary outcomes include a reliability index (Qualification Index) for BP epochs and count of qualified epochs.

**Results:**

Patient recruitment started in June 2021. Results are expected to be published by December 2021.

**Conclusions:**

The findings of the Senbiosys trial are expected to improve remote BP monitoring. The diagnosis and treatment of hypertension should benefit from these advancements.

**Trial Registration:**

ClinicalTrials.gov NCT04379986; https://clinicaltrials.gov/ct2/show/NCT04379986

**International Registered Report Identifier (IRRID):**

PRR1-10.2196/30051

## Introduction

Systemic arterial hypertension is a major modifiable cardiovascular risk factor [1]. The 10-year incidence of hypertension in the Swiss population has been estimated to be almost one-third [2]. Many patients with hypertension are aware of their condition but are either untreated or inadequately treated, although effective treatment of hypertension reduces morbidity and mortality rates [3].

Remote blood pressure (BP) monitoring—accompanied by education on hypertension management by caregivers—can substantially improve BP control. Wearable devices can provide user-friendly, accurate, and continuous BP monitoring to assess patient’s vital signs and achieve remote patient management [4-6].

The newest cuffless BP monitoring devices use photoplethysmography (PPG) [6,7]. This method consists of emitting light to the skin through a light-emitting diode and then measuring the changes in light absorption to a photodiode to detect peripheral volumetric variations of blood circulation [8]. A PPG waveform has been proven to correlate well with BP waveforms and truly represents one cardiac cycle, comprising the systolic peak, the diastolic peak, and the dicrotic notch [9,10]. One of the main challenges of this technique is to obtain an accurate estimate from the different PPG morphologies that are affected by patient’s characteristics, including age, vessel stiffness, cardiovascular disease, and other hemodynamic properties [11,12].

The Senbiosys device uses a PPG-based pulse wave analysis technique, which involves morphological analysis of the PPG pulse waveform thanks to an algorithm that identifies combinations of features of the PPG waveform to estimate BP [13,14]. Moreover, the PPG signal quality strongly depends on the body location [15]. In this regard, the Senbiosys PPG technology captures the PPG signal in the finger, which is known to be one of the best locations in terms of signal fidelity. The accuracy of the PPG-based technology is of fundamental importance, and several validation procedures for assessing the precision of BP monitoring devices have been developed [16].

The purpose of this study is to evaluate the accuracy of the Senbiosys device for measuring BP compared to invasive BP measurements with the arterial line, the gold standard in the hospital setting. Participants will undergo invasive BP estimation and will simultaneously wear the Senbiosys device.

## Methods

### Study Setting

This is a single-center, single-arm, prospective trial aiming to assess the accuracy of the Senbiosys device to estimate BP versus the invasive BP measurement. The patients will undergo invasive BP estimation and will simultaneously wear the device. This study will enroll 25 adult patients addressed for a coronarography and 10 patients in the intensive care unit (ICU). The intervention period that the devices are worn is between 10 and 15 minutes for each patient. There is no follow-up period after intervention.

### Inclusion and Exclusion Criteria

All patients 18 years or older either referred for coronarography or in the ICU requiring invasive BP monitoring and with an arterial catheter in place are eligible for the study. A modified Allen test will be routinely performed prior to arterial catheterization as per clinical routine.

The presence of any of the following exclusion criteria will lead to exclusion of the participants:

- Patient unable or unwilling to provide written informed consent themselves, which presupposes the patient’s capacity for discernment
- Coronarography in patients with myocardial infarction
- Patient with suspected or certified COVID-19 infection
- Patients with atrial fibrillation
- Patients with intracardiac monitoring
- Significant noninvasive systolic BP (SBP) difference between left arm and right arm (difference >20mmHg in systolic arterial pressure)

### Intervention

**Figure 1:**
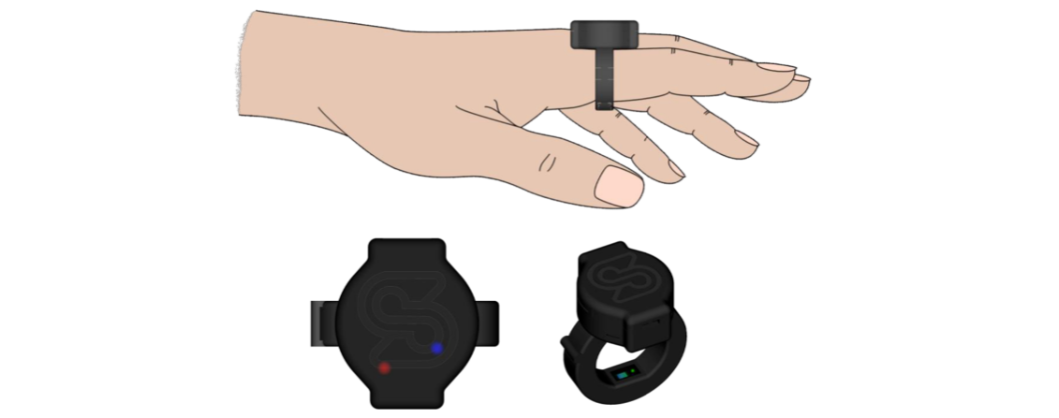
SBF2003 and the way the device is worn on a finger during operation.

The investigated device is the SBF2003, manufactured by Senbiosys, which is a ring measuring the patient’s PPG on the fingers. The intervention period that the devices are worn is between 10 and 15 minutes for each patient. The way the device is worn on a finger is depicted in Figure 1.

#### Standardized Measures in the Cardiac Catheterization Laboratory

The investigator will put the ring on the index finger of the patient’s hand. The device will be placed prior to the placement of the sterile field and will not be in contact with it. Arterial puncture will be performed either via the radial or femoral access according to the clinician’s choice. Intra-arterial BP waveforms will be recorded using a fluid-filled catheter. The catheter will be flushed before any waveform recordings are made. At first, the catheter will be positioned in the aorta for 3 minutes of stable BP waveforms recording. Intracoronary nitroglycerin will be administered at a dose of 300 µg. At the end of the coronary angiography, an additional 3 minutes of recording will be performed in the aorta.

#### Standardized Measures in the Intensive Care Unit

The investigator will put the ring on the same arm of the arterial catheter for simultaneous measurements. Enrolled patients must have had an arterial catheter in place at the time of inclusion to the study. No arterial catheters were placed for the sole purpose of this study. Arterial catheterization will be performed by the intensive care team according to current medical guidelines.

### Outcomes

The primary outcome is the assessment of mean bias (95% CI or precision of bias) for SBP, diastolic BP (DBP), and mean BP (MBP) between invasive and noninvasive BP measurements. The standard deviation of the bias (95% limits of agreement) will be assessed for SBP, DBP, and MBP measurements.

The percentage of signal with artifact, therefore not useable, will be determined. In this regard, secondary outcomes include a reliability index (Qualification Index) for BP epochs and count of qualified epochs.

### Participant Timeline

The timeline for enrollment, intervention, and data collection is outlined in Table 1.

**Table 1:** Timetable of patient enrollment and intervention and data collection.

Study schedule	Patient information and inclusion	Intervention period
Visit 1	0	1
Time	≥12h before procedure	0
Patient information	✓^a^	—^b^
Informed consent	✓	—
Baseline characteristics: age, gender, CVRF^c^, etc	✓	—
BP^d^ recordings	—	✓
Primary outcome	—	✓
Secondary outcome	—	✓
Safety outcome	—	✓

^a^This item is included.

^b^This item is not included.

^c^CVRF: cardiovascular risk factor.

^d^BP: blood pressure.

### Sample Size

The sample size is chosen based on the recommendations of the ISO 81060 guidelines [17] to determine the feasibility of noninvasive BP monitoring. Based on previous research, study sample size calculations for validation of BP measuring devices showed that 35 individuals is adequate for a high accuracy device (defined as mean BP difference between reference and test device measurement 0, SD 3-6 mmHg) [16]. Consensus was reached that studies performed on the general population should include adult participants; both individuals who are hypertensive and normotensive; ≥30% males and ≥30% females; ≥5% of the reference systolic BP readings ≤100 mmHg, ≥5% with ≥160 mmHg, and ≥20% with ≥140 mmHg; and ≥5% of reference diastolic BP readings ≤60 mmHg, ≥5% with ≥100 mmHg, and ≥20% with ≥85 mmHg. Due to the heterogenicity of patients to be included, we plan to enroll 25 patients in the cardiac catheterization laboratory and 10 patients in the ICU.

### Recruitment

Eligible participants will be identified via the principal investigator throughout the study period. The presence of exclusion criteria will be defined according to the patient’s clinician in charge. Patients’ capacity for discernment will be functionally assessed by the clinician in charge to determine whether the patient is capable of making a specific decision and whether the patient is capable of giving informed consent. If the eligibility criteria are met the patient will be formally enrolled. The written informed consent form will be signed by the patient and by one of the principal investigators. Only patients with the ability to provide consent in the ICU and the coronary care unit will be included.

Based on the current statistics for admissions to the ICU and cardiac catheterization laboratory in our institution, we estimate a total duration of 4 weeks for patient enrollment and the assessment of the primary and secondary end points.

### Data Collection

Patient characteristics and study outcomes will be transferred into an electronic case report form (REDcap Software, Vanderbilt University) designed to capture study information on the informatic structure of the HFR Fribourg. During the clinical trial, data will be accurately recorded, and the original documents will be stored at the clinical trials unit of the University and Hospital of Fribourg, under locked conditions when not in use.

### Statistical Methods

Categorical variables will be reported as counts and percentages; continuous variables will be reported as mean and SD or as median with 25% to 75% IQR according to their distribution as root mean square error. Normality will be assessed by visual inspection of histograms, the computation of Q-Q plots, and the Shapiro-Wilk test. Categorical variables will be compared using chi-square or Fisher exact test as appropriate. Continuous variables will be analyzed using the Student *t* test or the Wilcoxon rank sum test according to their distribution. Categorical variables fall into 3 groups representing the limit of the absolute BP differences: ≤5 mmHg, ≤10 mmHg, and ≤15 mmHg. Both arterial line and Senbiosys device signals will be segmented to epochs of duration (10-45 seconds). BP values will be computed for each epoch. Furthermore, for each epoch, we will compute the reliability index. Epochs with reliability indexes above a given threshold will be qualified for our study. Bland-Altman plots for repeated measures will be used to analyze SBP, DBP, and MBP data collected from the Senbiosys device and the arterial line [18]. Mean difference in scores (bias) and 95% limits of agreement, including the differences between noninvasive and invasive measurements (bias ± 1.96*SD), will be computed. Pearson correlation will be used to characterize the relation between noninvasive and invasive BP measurements. The acceptable bias and precision for arterial pressure measurements were fixed a priori at <5 and 8 mm Hg, respectively [16]. All statistical analyses will be performed using Matlab R2019a (MathWorks).

The null hypothesis is that the difference between invasive and noninvasive methods of BP calculated by Bland-Altman analysis are not within the clinically acceptable range.

### Analysis

All statistical analyses will be performed using Matlab R2019a. Blinded data analysis will be performed.

### Monitoring

The monitor is an independent trial monitor that will perform all the on-site monitoring activities. The monitor is qualified in the field of the International Standard through training and experience as well as scientific or clinical knowledge. The monitor will ensure that the clinical site understands and follows the protocol; he will review the completeness and accuracy of source data and study documents. Monitoring visits will be performed at the beginning, middle, and end of the study. Source data and documents are accessible to the monitor, and questions are answered during monitoring visits.

### Harms

Since the Senbiosys measurements will not be interpreted in the clinical context, there is no risk of misdiagnoses. Adverse device effect includes the risk of developing a cutaneous allergic reaction. However, this risk is minimal given that the equipment used is of the medical type. In that case, the sensor will be removed earlier, and the patient excluded from the trial. There are no foreseeable interactions with simultaneous medical interventions, as the medical device is on the index finger and does not interfere with other actions. The risk analysis and risk assessment are performed according to EN ISO 1497.

### Ethics and Dissemination

#### Research Ethics Approval

This study is conducted in compliance with the current version of the Declaration of Helsinki. The research project was approved by the local ethics committee of canton Vaud, Switzerland (CER-VD 2020-00996).

#### Protocol Amendments

The principal investigator is responsible for communicating important protocol modifications to the ethics committee and to the competent authorities, including the clinical trial registry (ClinicalTrials.gov NCT04379986).

#### Consent

The study nurse will collect patient information and provide a consent form with details on trial rationale, interventions, and potential benefits and harms. The patients will be given up to 24 hours to consider participation. Patients can withdraw their consent unconditionally, at any time, and without any justification. Medical data that have been collected to date will, however, be analyzed.

#### Confidentiality

The investigators will comply with local privacy laws. Anonymity of the participants will be guaranteed when presenting the data at scientific meetings or publishing them in scientific journals. Participants’ medical information obtained in this study is considered confidential, and disclosure to third parties is prohibited.

### Access to Data

Data will be stored physically and electronically on a secure central server at the clinical trials unit at the University and Hospital of Fribourg. Physical data are protected by restricted access to their location. Electronic data are protected by the IT-Services of the state of Fribourg (SITEL services). The investigators will have access to the protocol, data set, and statistical code during and after the study for publication and dissemination. The study nurse will only have access to the data set during the study period.

### Funding

The trial is supported by an unrestricted grant from the Fonds Scientifique Cardiovasculaire Fribourg.

### Dissemination Policy

The study results will be disseminated within the department of cardiology and the ICU, and are intended to be published in peer-reviewed medical journals and communicated at medical conferences.

## Results

This study was approved by the local ethics committee of the canton of Vaud, Switzerland (CER-VD 2020-00996) in March 2021. Patient recruitment started in June 2021. Data collection was completed by the end of June 2021. The results are expected to be published within 6 months of the end of the study.

## Discussion

During the last couple of years, a growing number of wearable devices evolved to provide accurate, low-cost, and easily applicable monitoring of vitals parameters using PPG [19], A recent study by Pellaton et al [20] compared SBP and DBP values obtained by radial artery catheterization and those obtained from optical measurements (PPG) at the wrist. Unlike this study, we aim at focusing on the finger as a better location for PPG extraction and consequently BP monitoring. The results of Pellaton et al [20] were quite promising and justifies a further exploration on the finger by the means of SBF2003.

By demonstrating that the Senbiosys SA technology is reliable, we aim to substantially improve BP monitoring. This is particularly relevant both for prevention and ambulatory monitoring. The diagnosis and treatment of hypertension are expected to largely benefit from these advancements.

## Abbreviations

BP: blood pressure
DBP: diastolic blood pressure
ICU: intensive care unit
MBP: mean blood pressure
PPG: photoplethysmography
SBP: systolic blood pressure
